# Validity of inferring size-selective mortality and a critical size limit in Pacific salmon from scale circulus spacing

**DOI:** 10.1371/journal.pone.0199418

**Published:** 2018-06-26

**Authors:** Terry D. Beacham, H. Andres Araujo, Strahan Tucker, Marc Trudel

**Affiliations:** Fisheries and Oceans Canada, Pacific Biological Station, Nanaimo, B. C, Canada; Texas A&M University, UNITED STATES

## Abstract

Size-selective mortality owing to lack of energy reserves during the first marine winter has been suggested to be a result of juvenile salmon failing to reach a critical size or condition by the end of their first marine summer and not surviving the following winter due to this presumed energy deficit. This hypothesis implies strong size dependency of mortality, and is subject to empirical data support for acceptance. Scale circulus spacing has been interpreted as an index for body size, and we reviewed the effect of size-selective mortality with a knife-edge mortality function on descriptive statistics for a scale circulus spacing index (SCSI). In order to invoke size selection as an important driver of mortality during the first year of ocean rearing, it is necessary to demonstrate not only that size-selective mortality is directed towards the smaller members of the population, but that the selective nature of the mortality can account for a substantial portion of the observed mortality. If the assumption is made that a random sample of a single juvenile population has been obtained, then studies that employ a SCSI to infer size-selective mortality coupled with a critical size limit must demonstrate a shift toward larger values of the SCSI, but also a concomitant reduction in the variance and range of the SCSI and an increase in the skewness and kurtosis of the SCSI values. Through simulation we found that the percentage of adults that displayed a SCSI value greater than the maximum observed in the juvenile sample was highly dependent on the initial juvenile sample size and size-selective mortality rate. Geographical distributions of juvenile Pacific salmon can be stratified by size, with larger individuals migrating earlier from local ocean entry locations than smaller individuals, and thus differential timing migration of juveniles based upon body size prior to the collection of the marine juvenile sample may be a more plausible explanation of published trends in the SCSI, rather than invoking substantial size-selective mortality and a critical size limit.

## Introduction

The role of juvenile body size in regulating mortality in marine fish has been an area of continuing interest [1], especially in Pacific salmon (*Oncorhynchus*). In particular, Beamish and Mahnken [2] have proposed that most natural mortality of Pacific salmon during the marine life history phase was size-dependent and occurs in two major episodes. The first phase of mortality was suggested to be predation based and occurs after the smolts enter the ocean (e.g. [3]), with other studies on salmonids typically reporting relatively high mortality after initial ocean entry [4, 5]. The second phase of mortality was suggested to occur in the fall and winter of the first year in the ocean, when those individuals that have not attained a critical size die because they are unable to meet minimum metabolic requirements [2]. Beamish et al. [6] indicated that Pacific salmon had to achieve a sufficient size by the end of the first marine summer to be able to survive the metabolic demands during a period of hypothesized energy deficit in the late fall and winter.

In salmon, the critical-size hypothesis has been primarily tested using indices of size and growth derived from scales collected on salmon during their first year at sea and on returning adults. For instance, in a study based upon scale circuli spacing, Beamish et al. [6] reported that coho salmon (*O*. *kisutch*) that survived their first winter at sea had significantly larger spacing between circuli on scales, indicating that brood year strength was related to growth in the first marine year. As a result, they suggested that size-related mortality in the first marine fall and winter was an important determinant of brood year strength of some coho salmon stocks and stocks of other species of Pacific salmon, and that individual coho salmon needed to attain a critical minimum size by the fall of their first year at sea in order to survive the subsequent winter. Moss et al. [7], in a study based on scale circulus spacing in pink salmon (*O*. *gorbuscha*), suggested that at the same circulus, a significantly larger average scale radius for returning adults than for juveniles from the same hatchery would suggest that larger, faster-growing juveniles had a higher survival rate and that significant size-selective mortality occurred after the juveniles were sampled. Moss et al. [7] noted that the results supported other studies that found that larger, faster-growing fish were more likely to survive until maturity, and were indicative of a critical minimum size threshold. Zavolokin and Strezhneva [8], in a study on pink salmon, reported confirmation of a critical size and critical period hypothesis, whereby smaller individuals that did not accumulate enough energy reserves during the first summer were disproportionately eliminated during the subsequent winter compared with larger, faster-growing individuals. Similar results have been reported by other investigators for Chinook salmon (*O*. *tshawytscha*) [9, 10] and steelhead (*O*. *mykiss*) [11]. Thus, there is a belief that size-selective mortality during the first year of ocean rearing is the prime driver of salmon survival and recruitment, and that there is a critical size necessary for salmon to obtain by the fall of the first marine year in order to survive the winter [12, 13].

Yet in recent studies of juvenile coho salmon size variation, we could find no evidence of any critical size that age 1.0 coho salmon juveniles in southern British Columbia had to attain by the end of the first summer or fall of marine rearing to enable them to survive the subsequent winter in the ocean [14]. There was no support for the hypothesis that Pacific salmon had to achieve a sufficient size (the “critical” size) by the end of the first marine summer or fall to be able to survive during the winter of their first year of ocean rearing. Instead, our work suggested that larger age 1.0 coho salmon had left their early marine areas earlier. If the adult sample was derived from two juvenile populations, the first population comprised of smaller sampled individuals, and the second population comprised of larger unsampled individuals, then possibly unrepresentative sampling of the juvenile population may account for observed trends in descriptive statistics of the adult distribution. These findings caused us to evaluate salmon studies in which marked size-selective mortality coupled with a critical size was reported over the first winter of marine rearing. Attempting to reconcile this observation with current theory led us to reflect on some fundamentals in terms of sampling theory, proper sampling design and sample sizes and appropriate analytical steps.

If evaluation of differences in frequency distributions of individuals with different scale circulus spacing is a valid way to infer size-selective mortality of juveniles, then a number of predictions can be made with respect to the distribution of individuals with different circulus spacing within the population. First, there should be a positive correlation between circulus spacing and body size both within and among sampling periods, as individuals with wider circulus spacing are imputed to be faster growing and thus larger. Next, if circulus spacing is a permanent record of growth, there should be a wide range of circulus spacing within the population of juveniles sampled during the first marine summer, but during the late fall and winter as hypothesized size-selective mortality occurs, individuals with narrow circulus spacing should disappear from the population as they were unable to attain the critical size necessary for survival during the first winter of ocean residence. Given an appropriate sample design and size, there should be little identification of individuals later in the life cycle with circulus spacing unobserved during the first summer of rearing, merely a change in the relative frequencies of circulus spacing observed in the juvenile population during the first summer of rearing. Therefore, one should expect that the range of circulus spacing in the population should be greater during the first summer of marine rearing than later in the life cycle, and that the variance in circulus spacing within the population should be greater in the first summer than later in the life cycle.

In this study, we evaluate the hypothesis of size-selective mortality of juvenile salmonids as inferred by changes in frequencies of scale circulus spacing, coupled with the attainment of a critical size. First, we construct a population of juveniles with a scale circulus spacing index (SCSI) normally distributed within the population, as would be expected to be observed during sampling of juveniles during the first summer of ocean rearing. We then apply varying scenarios of size-selective mortality rates, examining the resultant descriptive statistics of the distribution in order to evaluate distributions observed in published studies. Second, if the adult sample is derived from two juvenile populations, the first population comprised of smaller sampled individuals, and the second population comprised of larger unsampled individuals, we examine the descriptive statistics of the adult distribution. Finally, we compare the results of previously published studies and evaluate whether the descriptive statistics support a size-selective mortality coupled with a critical size limit hypothesis, or a second unsampled juvenile population of larger individuals hypothesis.

## Results

### Expected distribution of scale index values for adults

A normally distributed juvenile population with the SCSI displaying a mean of 30 and a standard deviation of 5 was subjected to simulated size-selective mortality with a critical size limit under the assumption that a critical size had to be obtained in order to survive to the adult stage. Size-selective mortality rates of 20%, 40%, 60%, and 80% coupled with a critical size limit were applied to the juvenile population prior to the collection of simulated adult samples. As the simulated juvenile mortality rate increased, the median SCSI of the adult sample increased, the range of the SCSI declined, as did the standard deviation (SD) of the SCSI (Table 1). The kurtosis and skewness of the distribution of the SCSI values of the adults increased positively, with higher values observed under more extensive mortality scenarios (Table 1). Importantly, as it was assumed that the SCSI value for an individual was fixed during the life cycle, the percentage of adults that displayed a SCSI value greater than the maximum observed in the juvenile sample was highly dependent on the initial juvenile sample size and size-selective mortality rate (Table 1). The proportion of adults that displayed a SCSI value greater than observed in the juvenile sample was larger at the higher size-selective mortality rates and smaller juvenile sample sizes relative to adults. If only 50 juveniles were sampled, up to 18% of the adults sampled could be larger than the largest individual in the juvenile sample. However, when at least 200 juveniles were sampled, even when the size-selective mortality rate coupled with a critical size limit was 80% and adult sample sizes were up to five times larger than that of juveniles, the proportion of adults that displayed a SCSI value greater than observed in the juvenile sample was < 4.5%. If the juvenile sample size was 1,000 individuals, the percentage of adults that displayed a SCSI value greater than observed in the juvenile sample was ≤ 1%.

**Table 1:** Median, range, range minimum (Min), range maximum (Max), standard deviation (SD), kurtosis, and skewness (Skew) of a scale circulus width index in a simulated adult sample where only a single juvenile population has contributed to the adult sample. The juvenile population displayed a mean of 30, SD of 5, and skewness and kurtosis of 0.00 for the index. Size-selective mortality rates (20%-80%) coupled with a critical size limit were applied to the smaller members of the juvenile population. Percentage (%) of individuals in the adult sample indicates the median index value of the percentage of individuals in the adult sample that were larger than the largest value observed in the juvenile sample. The sample size from the juvenile population was either 50, 200 or 1000 individuals, with the subsequent adult sample size ranging from 50 to 1,000 individuals.

Juvenile	Adult	Mortality	Median	Range	Min	Max	SD	Kurtosis	Skew	%
50	50	20	30.5	10.7	26.0	36.7	2.6	-0.5	0.4	2.0
		40	31.6	8.3	28.8	37.1	2.0	-0.2	0.7	2.0
		60	33.2	6.5	31.3	37.8	1.6	0.3	1.0	4.0
		80	35.6	5.0	34.2	39.2	1.2	0.8	1.2	18.0
	200	20	30.5	12.3	25.9	38.2	2.6	-0.3	0.4	1.5
		40	31.6	10.0	28.8	38.5	2.0	0.2	0.8	2.0
		60	33.3	7.8	31.3	39.1	1.6	1.0	1.1	4.0
		80	35.6	6.2	34.2	40.4	1.2	1.8	1.3	17.5
	600	20	30.5	13.4	25.8	39.2	2.6	-0.2	0.4	1.5
		40	31.7	10.8	28.7	39.5	2.0	0.4	0.8	2.2
		60	33.3	8.8	31.3	40.1	1.6	1.2	1.1	4.0
		80	35.6	7.1	34.2	41.3	1.2	2.1	1.4	16.8
	1000	20	30.5	13.8	25.8	39.6	2.6	-0.2	0.4	1.4
		40	31.5	11.2	28.7	39.9	2.0	0.4	0.8	2.1
		60	33.3	9.2	31.3	40.5	1.6	1.3	1.1	4.2
		80	35.6	7.4	34.2	41.6	1.2	2.2	1.4	17.1
200	50	20	30.5	10.7	26.0	36.7	2.6	-0.5	0.4	0.0
		40	31.6	8.3	28.8	37.1	2.0	-0.2	0.7	0.0
		60	33.3	6.5	31.3	37.8	1.6	0.3	1.0	0.0
		80	35.6	5.0	34.2	39.2	1.2	0.8	1.2	4.0
	200	20	30.5	12.3	25.9	38.2	2.6	-0.3	0.4	0.5
		40	31.6	9.7	28.8	38.5	2.0	0.2	0.8	0.5
		60	33.3	7.9	31.3	39.2	1.6	1.0	1.1	1.0
		80	35.6	6.2	34.2	40.4	1.2	1.8	1.3	4.5
	600	20	30.5	13.4	25.8	39.2	2.6	-0.2	0.4	0.3
		40	31.5	10.7	28.7	39.4	2.0	0.4	0.8	0.2
		60	33.3	8.8	31.3	40.1	1.6	1.2	1.1	1.0
		80	35.6	7.1	34.2	41.3	1.2	2.1	1.4	4.3
	1000	20	30.5	13.8	25.8	39.6	2.6	-0.2	0.4	0.4
		40	31.6	10.5	28.7	39.2	2.0	0.4	0.8	0.5
		60	33.3	9.2	31.3	40.5	1.6	1.3	1.1	1.0
		80	35.6	7.4	34.2	41.6	1.2	2.2	1.4	4.3
1000	50	20	30.5	10.7	26.0	36.7	2.6	-0.5	0.4	0.0
		40	31.6	8.3	28.8	37.1	2.0	-0.2	0.7	0.0
		60	33.2	6.5	31.3	37.8	1.6	0.3	1.0	0.0
		80	35.6	5.0	34.2	39.2	1.2	0.9	1.2	0.0
	200	20	30.5	12.3	25.9	38.2	2.6	-0.3	0.4	0.0
		40	31.7	9.7	28.8	38.5	2.0	0.2	0.8	0.0
		60	33.3	7.8	31.3	39.1	1.6	1.0	1.1	0.0
		80	35.6	6.2	34.2	40.4	1.2	1.8	1.3	1.0
	600	20	30.5	13.4	25.8	39.2	2.6	-0.2	0.4	0.0
		40	31.6	10.8	28.7	39.5	2.0	0.4	0.8	0.0
		60	33.3	8.8	31.3	40.1	1.6	1.2	1.1	0.2
		80	35.6	7.1	34.2	41.3	1.2	2.1	1.4	0.8
	1000	20	30.5	13.9	25.8	39.7	2.6	-0.2	0.4	0.1
		40	31.6	11.3	28.7	40.0	2.0	0.4	0.8	0.1
		60	33.3	9.2	31.3	40.5	1.6	1.3	1.1	0.2
		80	35.6	7.4	34.2	41.6	1.2	2.2	1.4	0.9

The effect of two juvenile populations contributing to the sample of adults was investigated with respective to the trends in descriptive statistics outlined previously. With the first juvenile population displaying a mean of 30 and a SD of 5 for the SCSI distribution, and the second population displaying a mean of 35 and a SD of 5 or a mean of 40 and a SD of 5, the effect of differing population contribution rates to the adult sample was investigated. As the contribution of the second population to the simulated adult sample increased, the median SCSI of the adult sample increased, the range of the SCSI generally increased, and the SD of the SCSI increased (Table 2). The kurtosis and skewness of the distribution of the SCSI values of the adults both declined, with lower values observed under more extensive contributions from the second population with the higher mean SCSI value. The percentage of individuals in the adult sample that displayed a SCSI value greater than the maximum observed in the juvenile sample from population 1 increased as the contribution from population 2 increased, regardless of the size of the juvenile and adult samples (Table 2). For example, up to approximately 34% of the adult sample could display a SCSI value greater than that observed in the corresponding juvenile sample from population 1, dependent upon the contribution rate of the second juvenile population to the adult sample and the size of the juvenile sample.

**Table 2:** Median, range, range minimum (Min), range maximum (Max), standard deviation (SD), kurtosis, and skewness (Skew) in a simulated adult sample where two juvenile populations have contributed to the adult sample. Juvenile population 1 displayed a mean of 30 and SD of 5, population 2 displayed a mean of 35 and SD of 5, and population 3 displayed a mean of 40 and SD of 5. Percentage (%) of individuals in the adult sample indicates where the scale index value was > the largest value observed in the juveniles sampled from population 1. Juvenile population 1 contributes 90% to 40% of the adult sample, and either juvenile population 2 or 3 contributes the remainder. Adult sample size was 50 and 200 individuals.

Pop 1	Pop 2	Pop 3	Mean	Range	Min	Max	SD	Kurtosis	Skew	%
Sample size 50										
90	10		30.4	22.9	19.1	42.0	5.2	-0.3	0.1	0.0
80	20		30.9	23.6	19.4	43.0	5.3	-0.3	0.1	2.0
70	30		31.4	24.1	19.6	43.7	5.5	-0.3	0.1	4.1
60	40		32.0	24.4	19.9	44.3	5.6	-0.4	0.0	6.0
50	50		32.5	24.4	20.3	44.7	5.6	-0.4	0.0	8.0
40	60		33.0	24.4	20.7	45.1	5.5	-0.4	0.0	12.0
90		10	30.8	26.2	19.1	45.3	5.7	-0.0	0.3	4.1
80		20	31.8	27.9	19.4	47.3	6.3	-0.2	0.4	8.2
70		30	32.9	28.7	19.7	48.4	6.7	-0.5	0.3	12.2
60		40	34.0	29.1	20.0	49.1	7.0	-0.7	0.1	20.0
50		50	35.0	29.2	20.4	49.6	7.1	-0.7	-0.0	26.0
40		60	36.0	29.2	20.8	50.0	7.0	-0.7	-0.1	34.0
Sample size 200										
90	10		30.5	28.3	16.7	45.0	5.2	-0.1	0.1	0.5
80	20		31.0	29.0	16.9	45.9	5.4	-0.1	0.1	1.0
70	30		31.5	29.4	17.1	46.5	5.5	-0.1	0.1	1.5
60	40		32.0	29.7	17.3	47.0	5.6	-0.2	0.0	2.5
50	50		32.5	29.9	17.6	47.5	5.6	-0.2	-0.0	3.5
40	60		33.0	29.9	18.0	47.9	5.6	-0.2	-0.0	5.0
90		10	30.9		16.7	49.0	5.8	0.2	0.4	4.5
80		20	32.0		16.8	50.5	6.4	-0.0	0.4	5.0
70		30	33.0		17.1	51.4	6.8	-0.3	0.3	8.0
60		40	34.0		17.4	51.9	7.0	-0.5	0.1	11.5
50		50	35.0		17.7	52.3	7.1	-0.6	0.0	16.0
40		60	36.0		18.1	52.6	7.0	-0.6	-0.2	21.0

**Fig 1:**
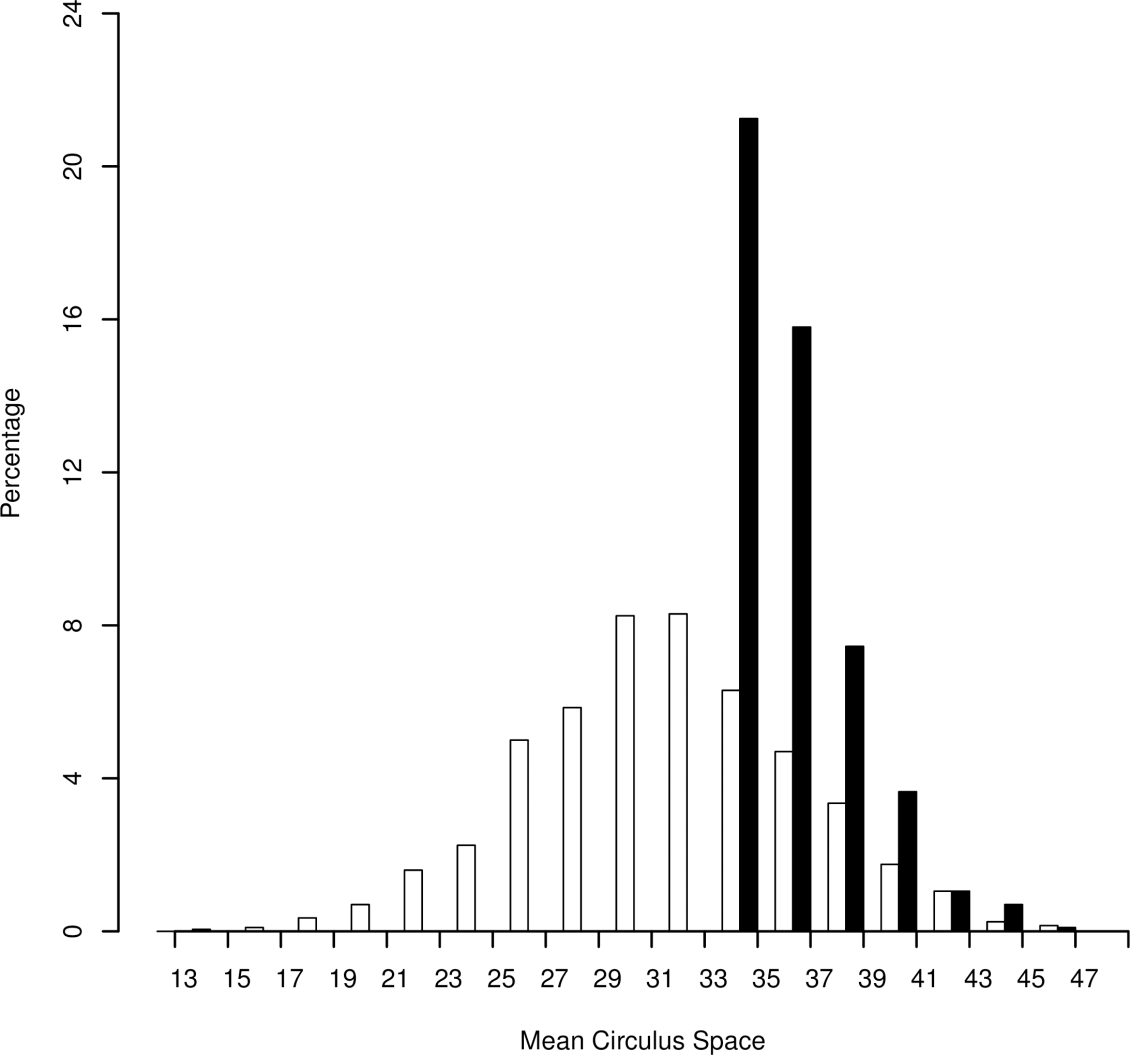
Histograms of scale circulus spacing index values where the juvenile population displayed a mean of 30, SD of 5, and skewness of 0.00 for the index, with the juvenile sample comprised of 1,000 individuals (open bars). The adult sample (black bars) of 1,000 individuals was derived by applying size-selective mortality coupled with a critical size limit to the smaller members of the juvenile population, with potential juveniles discarded from the adult sample if they originated from the smaller 80% of the juvenile population.

Two radically different hypotheses, that of size-selective mortality coupled with a critical size limit versus two juvenile populations contributing to the adult sample both result in an increased mean SCSI value and a shift to the right in a histogram of the distribution of adult SCSI values relative to the juveniles. However, these hypotheses produce markedly different trends in other descriptive statistics. Under the size-selective mortality and critical size limit hypothesis, where both the sampled juveniles and adults are derived from a single population, the range and SD of the SCSI values should decline in the adult sample, skewness and kurtosis increase, and most adults should display SCSI values within the range observed in the juvenile sample. Now if representative samples of the both the juvenile and adult populations have been obtained, the scale index is fixed over the life of an individual, and size-selective mortality rates are substantial and based upon attainment of a critical minimum size, we suggest that the distribution of the adult scale index relative to the juvenile index should resemble that displayed in Fig 1.

**Fig 2:**
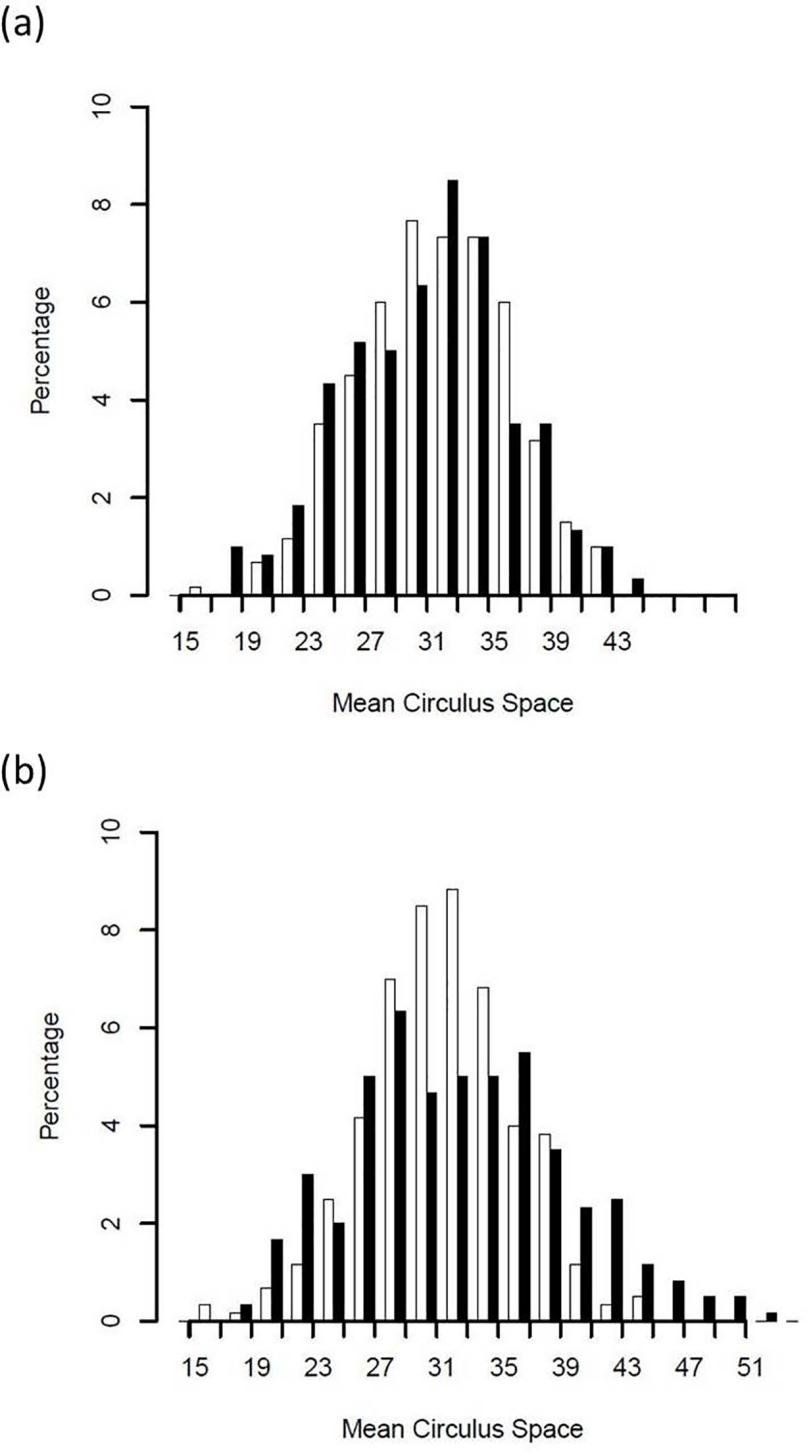
(a) Histograms of scale circulus spacing index values where juvenile population 1 displayed a mean of 30, SD of 5, and skewness of 0.00 for the index, with the juvenile sample comprised of 200 individuals (open bars). The adult sample (black bars) of 200 individuals was derived by having 70% of the sample derived from juvenile population 1 and 30% from juvenile population 2 with a mean of 35 and SD of 5. (b) Same conditions as (a), except juvenile population 2 displayed a mean of 40.

In contrast, if the adult sample is derived from two juvenile populations, the first population comprised of smaller sampled individuals, and the second population comprised of larger unsampled individuals, then the opposite trends in descriptive statistics (other than increasing mean) should be observed in the adult sample. If the distribution of the adult scale index relative to the juvenile index is similar to that displayed in Fig 2A and 2B, what inferences can be drawn with respect to the effect and magnitude of size-selective mortality? Evaluation of this question was the central theme of the current study.

## Discussion

We now examine some studies that concluded size-selective mortality concentrated on the smaller members of the populations was important in subsequent population abundance in relation to expected trends in the descriptive statistics outlined previously.

### Evaluation of previous studies: Juvenile sampling in fresh water

Bond et al. [15] compared the size distributions of hatchery-reared steelhead (*O*. *mykiss*) smolts sampled immediately before release with the back-calculated size at ocean entry of surviving adults from the same cohort. Size was back-calculated with a scale radius and fork length regression, with an intercept of 34, which would be interpreted as the fork length (mm) at initial scale formation. All adults in the sample displayed back-calculated fork lengths that were observed in the smolts upon release, and the back-calculated lengths were a subset of those observed in the smolts, with a shift in the frequency distribution of size towards values associated with larger individuals. These results were consistent with those that were observed in our sampling example and illustrate results that would be expected if the smaller individuals in the smolt population experienced size-selective mortality. We suggest that the key here was that the juvenile samples were representative of the entire population, as the juveniles were sampled before hatchery release, and the returning adults must necessarily have been derived from the sampled juvenile population. There was presumably limited straying from other populations.

Thompson and Beauchamp [11] evaluated size distributions of steelhead (*O*. *mykiss*) juveniles, smolts, and adults, with juvenile lengths compared with back-calculated surviving smolt and adult lengths at the first, second, and third freshwater annuli, with lengths calculated with a scale radius and fork length regression. In contrast to the results of [15], a portion of the back-calculated lengths of smolts and adults was larger than those ever observed in the juvenile population, with the portion never observed in the juvenile population increasing at each successive annulus. There was no compression of the range for the SCSI value observed, and no reduction in variance. About 22% of the adult population displayed back-calculated fork lengths at the third freshwater annulus greater than ever observed in the comparable juvenile cohort with three freshwater annuli.

### Evaluation of key previous studies: Juvenile sampling in marine waters

**Fig 3:**
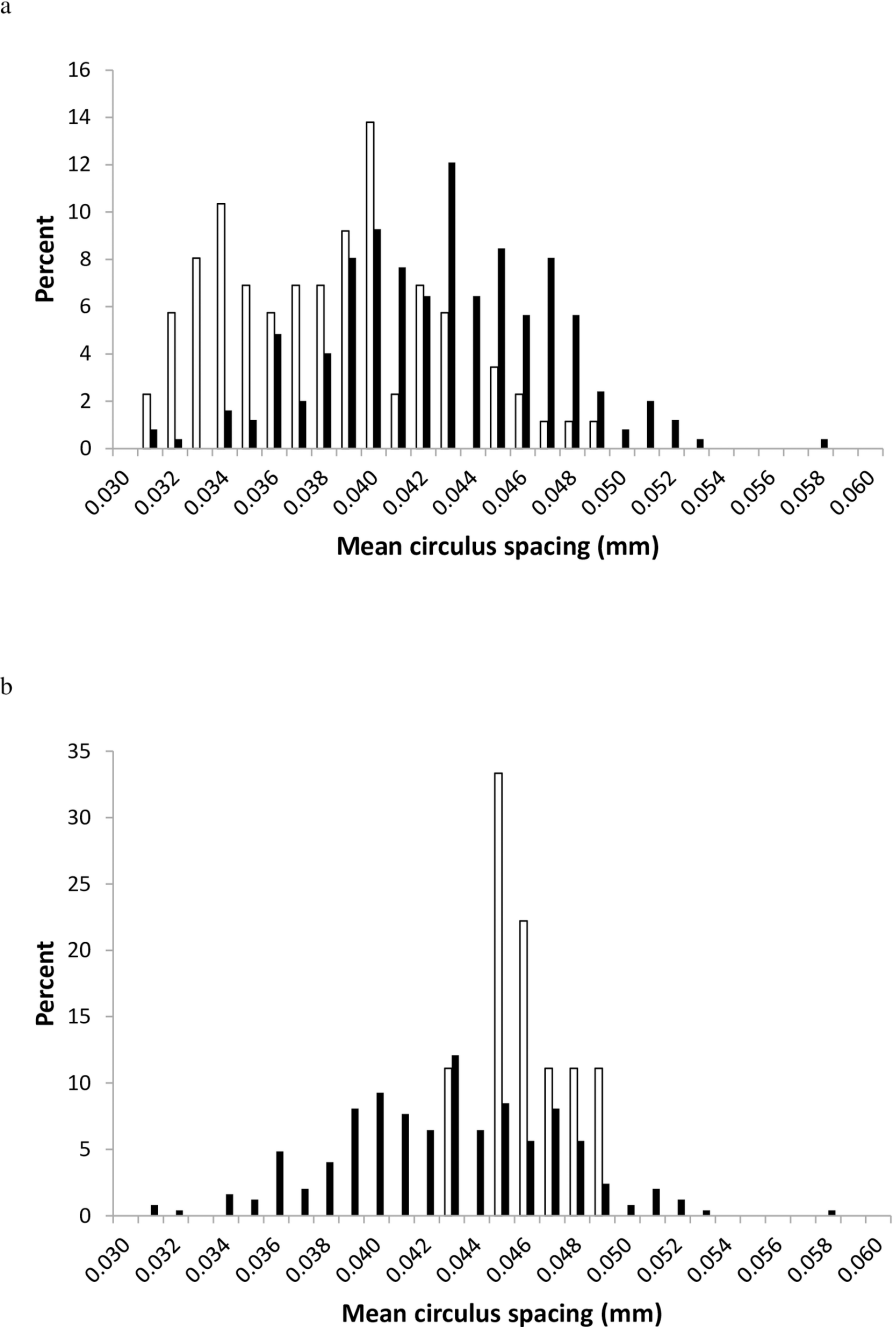
a. Observed distribution of mean circulus spacing of the first 10 marine circulus for marine age-0 coho salmon collected in the Strait of Georgia during September and November 2000 (open bars) and marine age-1 individuals collected from March through spawning 2001 (black bars)(from [6]). b. Projected distribution of mean circulus spacing for age-0 individuals shown in 3a under the assumption of a subsequent size-selective mortality rate of 90% (open bars) and actual distribution (black bars) observed from sampling age-1 individuals in 2001.

Many investigations proceed by sampling juveniles after they had been rearing for a period of time in the ocean. Beamish et al. [6] measured mean intercirculus spacing of the first 10 marine circuli of coho salmon in the Strait of Georgia in British Columbia, with juveniles sampled in September and November of their first year of ocean rearing (marine age 0). Intercirculus spacing of marine age 1 individuals sampled in the following year was then compared with the juvenile sample, and size-selective mortality was inferred, as the distribution of adult intercirculus spacing was shifted to the right of the juvenile distribution. If size-selective mortality was indeed acting upon the smaller members of the population below a critical minimum size, then the range and variance of the adult circulus spacing values should decline, skewness of the distribution should increase (Table 1), and most adults in the sample should display circulus width values observed in the juvenile population. The results reported by Beamish et al. [6] in their Fig 1 in fact display the opposite results to those expected under a size-selective mortality environment directed at the smaller members of the population. The range and variance of the scale index values of the marine age-1 individuals were actually larger than those of the age-0 individuals and skewness of the distribution declined (Table 3), with most characters indicative of relatively more marine age-1individuals with narrow circulus width spacing than would be expected. If the 90% mortality rate suggested by Beamish et al. [6] was size selective against the smaller individuals, the expected distribution of the circulus width values was quite different than that of the observed distribution (Fig 3), which does not provide support for significant size-selective mortality for the smaller members of the population. Instead, Beacham et al. [14] suggested that the September and particularly the November circulus spacing distributions analyzed by Beamish et al. [6] were likely not representative of the whole juvenile population and underestimated the presence of the larger coho salmon that were resident in the ocean but unavailable to the fall sampling regime reported by Beamish et al. [6], resulting in an overestimation of the impact of size-selective mortality on the population.

**Table 3:** Mean, range, range minimum, range maximum, standard deviation, kurtosis, and skewness of juvenile and immature coho salmon mean intercirculus distance (mm) for the first 10 marine circulus as outlined in Fig 1 of [6]. Sample 1 originated from ocean age-0 juveniles sampled in the Strait of Georgia in 2000. Sample 2 originated from ocean age-1 immature individuals sampled in the Strait of Georgia during 2001, as well as some mature individuals sampled at two hatcheries.

	Sample 1	Sample 2
N	87	248
Mean	0.038	0.043
Range	0.018	0.027
Range minimum	0.031	0.031
Range maximum	0.049	0.058
Standard deviation	0.004	0.004
Kurtosis	-0.43	0.15
Skewness	0.40	0.04

The study of Moss et al. [7] has been described as the “gold standard” in conducting direct comparisons of distributions of scale characters of juveniles and adults (E. Farley, U.S. National Marine Fisheries Service, Juneau, Alaska, personal communication), with inferences drawn concerning size-selective mortality between the juvenile and adult life history stages. Moss et al. [7] measured distances (μm) from the scale focus to a specific circulus for three hatchery populations of pink salmon in Prince William Sound, Alaska. Size-selective mortality was inferred by comparing the frequency and means of scale radius length classes at a specific circulus for juveniles from the hatcheries sampled in Prince William Sound and the Gulf of Alaska during July, August, and September in the year of release with that of adults that returned to the three hatcheries the following year. As noted previously, if substantial size-selective mortality were present between the juvenile and adult life history stages as reported by Moss et al. [7], then the majority of adults in the sample should display SCSI values observed in the juvenile population (Table 1), and the range and variance of the adult circulus spacing values should decline. The results presented in Fig 2 of Moss et al. [7] for pink salmon from the Armin F. Koernig (AFK) hatchery at circulus 15 indicated that approximately 58% of the adults displayed scale radius lengths greater than ever observed in the comparable marine age-0 juvenile population, with this discrepancy being significant (χ^2^ = 31.3, df = 1, P<0.01). Similarly, approximately 18% of adults from the Cannery Creek (CC) hatchery displayed scale radius lengths greater than ever observed in the marine age-0 juvenile population (χ^2^ = 6.4, df = 1, P<0.05), and approximately 46% of adults from the Wally Noerenberg (WN) hatchery displayed scale radius lengths greater than ever observed in the marine age-0 juvenile population (χ^2^ = 15.6, df = 1, P<0.01). These results were similar to those expected under the hypothesis of an unsampled juvenile population comprised of larger individuals than those observed in the sampled juvenile population contributing substantially to the adult sample (Table 2). With respect to range of scale radius lengths to circulus 15, adults from the three hatcheries displayed a range of 55 μm to 135 μm more than the juveniles at the respective hatcheries. Contrary to expectations under a hypothesis of size-selective mortality directed at the smaller members of the population, the range of circuli spacing in the adult populations was actually greater than observed in the respective juvenile populations from the previous year, and a substantial portion of the adults displayed scale radius lengths greater than ever observed in the comparable marine age-0 juvenile population. Similar to the results of [6], it seems likely that the juveniles sampled were not representative of the whole population and underestimated the presence of the larger pink salmon that were resident in the ocean but unavailable to the summer and fall sampling regime reported by Moss et al. [7], resulting in an overestimation of the impact of size-selective mortality on the populations.

Zavolokin and Strezhneva [8] measured scale increments in juvenile pink salmon that were caught in the southern Sea of Okhotsk in the fall of 2007 and 2008 and subsequently maturing individuals in the summer of 2008 and 2009. They concluded that the hypothesis of a critical size and a critical period (the winter) was confirmed, based in part upon their interpretation that in the 2007 year class, the average scale increment to the 12^th^ circulus in the fall juvenile sample (n = 1122, mean = 459, SD = 59) was smaller than in the subsequent summer sample of maturing adults (n = 329, mean = 564, SD = 72). However, the SD was larger in the adults than in the juveniles, and approximately 11% of the maturing adults displayed an increment larger than ever observed in the juvenile population, a value far higher than expected with a juvenile sample size in excess of 1,100 individuals (Table 1). The range of increments for the juvenile population spanned 300 units, as did the maturing adults. There was an increase in the mean, no reduction in the range, and in fact an increase in variance for the scale increment in the adult sample as compared with the juvenile sample. Thus, as trends in range and variance of the scale increment did not conform to those expected under size-selective mortality with a critical size limit (Table 1), confirmation of a critical size and critical period is questionable. Interestingly, for the 2008 year class, the adult sample (n = 418) displayed an absence of adults in the larger SCSI values observed in the juveniles, resulting in a reduction in the mean (649 vs 587), reduction in standard deviation (98 vs 64), and reduction in range (550 vs 400) compared with the juvenile sample (n = 476), all trends that would be expected if there were size-selective mortality against the larger members of the juvenile population, not the smaller members. However, Zavolokin and Strezhneva [8] interpreted these results to conclude that the distribution of SCSI values of juveniles and adults in the 2008 year class was “similar” and provided no evidence of size-selective mortality.

Howard et al. [10] reported size-selective mortality of Yukon River Chinook salmon (*O*. *tshawytscha*), based upon sampling of over 1,000 juveniles in the eastern Bering Sea, sampling over 800 returning adults at a test fishery in the lower river to obtain scales, back calculating length to be comparable with juveniles via a relationship between scale radius and body length, and determining a weight distribution from the lengths via a length-weight relationship. Three percent of the juvenile weight distribution was lower than that ever observed in the returning adults, and 6% of the returning adults were larger than ever observed in the juvenile weight distribution. The range of the juvenile weight distribution was approximately 230 g, while that back calculated for the returning adults at the comparable time was 260 g. Qualitatively, these results were very similar to those reported by [6] for coho salmon in the Strait of Georgia, and display the same non-conformance to expected results if indeed size-selective mortality were a significant component of mortality between juvenile sampling and returning adults. In particular, there was an increase in the range of weights back calculated for returning adults relative to the juveniles, as well as an increase in variance of weight, unexpected if both juveniles and adults were derived from a single population (Table 1). A portion of the returning adults was larger than would be expected given the juvenile sample size in excess of 1,000 individuals (Table 1), and thus the juvenile weight distribution analyzed by [10] was likely not representative of the whole stock and underestimated the presence of the larger juvenile Chinook salmon that were resident in the ocean but unavailable to the trawl sampling. In addition, although Yukon River juvenile abundance during the first marine summer prior to any winter mortality has been reported to be highly correlated with subsequent adult abundance [16], Howard et al. [10] stated that the first winter in the ocean was a second critical period for Yukon River chinook salmon, regardless of the observation that the critical period hypothesis required that the mortality be important in determining year-class abundance [2]. The authors had no way of determining the level of mortality or partitioning mortality during the first winter from mortality experienced in subsequent years prior to maturity (up to six years).

The results outlined by [6], [7], [8], [10], and [11] were qualitatively different than those of [15], and all groups of authors interpreted their results to indicate that size-selective mortality directed against the smaller juveniles accounted for the differences in distribution of the circulus index between juveniles and adults. However, in all four studies where marine samples of juveniles were obtained, there was no reduction in the range and variance of the adult SCSI, and adults were observed or estimated to be present that had SCSI values larger than the upper end of the range observed in the juvenile population. If the SCSI patterns investigated were fixed early in juvenile marine rearing, the fundamental question to answer is how is it possible that adults derived from the juvenile population displayed values greater than the maximum observed in the juvenile population. Our simulated juvenile and adult samples suggested that a maximum of 4.5% of the adult sample may be comprised of individuals larger than the largest sample juvenile if 200 juveniles had been sampled and the juveniles and adults comprise a single population. In the studies previously reviewed where a marine sample of the juveniles was obtained, 5%-58% of the adults sampled displayed a SCSI value larger than the largest juvenile sampled. One possible explanation is that the larger-sized individuals in the juvenile population were not available to be sampled when the initial distribution of the SCSI values in the juvenile population was determined. The larger-sized individuals could have selectively moved from the geographic sampling area [14, 17, 18, 19, 20], or they could have moved deeper in the water column making them unavailable to the sampling gear. It is possible that larger-sized juveniles were not available to be sampled as juveniles, but were available to be sampled as adults, resulting in observed SCSI values outside of the range observed in juveniles (Table 2). If so, then the reported size-selective mortality directed against smaller members of the population reported by [6], [7], [8], [10], and [11] may be plausibly explained as movement of larger-sized juveniles from the marine sampling areas prior to the collection of samples defined to be from the first ocean year individuals [21]. Geographical distributions of juvenile Pacific salmon are stratified by size, with larger individuals migrating earlier from local ocean entry locations than smaller individuals [14, 17, 18, 19, 20, 22, 23], and thus differential timing migration of juveniles based upon body size prior to the collection of the juvenile sample may be a more plausible explanation of observed trends in scale parameters, rather than invoking substantial size-selective mortality and a critical size that the juveniles had to achieve by the end of the first marine summer or fall to be able to survive during the winter of their first year of ocean rearing. This interpretation is also in line with assumptions of techniques underpinning these studies; the application of scale circulus spacing as a proxy for size.

Biased sampling of the juveniles may be a plausible explanation for how individuals with scale circuli values unseen in the juvenile population can be present in the adult population. It may also be possible that the assumption that scale circuli spacing values were fixed early in marine rearing and constant over an individual’s life may not be valid. The key question to answer is if one measured the distance from the scale focus to a specific circulus, such as marine circulus 10 for Beamish et al. [6] and circulus 15 for Moss et al. [7], would this distance be constant during the life cycle of an individual as assumed by [6]? In the study of [24], juvenile coho salmon were reared for a period of time in tanks, anesthetized, fork length recorded, individuals individually marked, and scales taken from the preferred area. However, Fisher and Pearcy [24] presented no evidence that scale growth and circuli spacing during the period prior to the start of the experiment were a permanent record of growth. Friedland and Haas [25] and Friedland and Redding [26] measured circuli spacing in Atlantic Salmon, but measurements were never taken on scales from the same individual at different times in the life cycle, so there was no evidence presented that illustrated that early marine circuli spacing was constant over the life cycle. Relative circuli spacing was different among groups of salmon, but there was no indication that the absolute spacing was fixed during early marine rearing.

If early marine circulus spacing is related to body size and is a fixed character during the life cycle of an individual, then it is reasonable to use circulus spacing as a proxy for body size, with individuals having wider spacing presumably growing faster and thus of larger inferred body size. However, wider scale circulus spacing may not necessarily be an indication of higher growth rates. Marco-Ruis et al. [27] reported that sea trout (*Salmo trutta*) that emigrated to sea after only one year of growth in freshwater were faster growing and tended to have smaller average inter-circulus spacing during the first months of life compared with those individuals that delayed migration for one or two additional years. They reported that this was largely a consequence of faster-growing individuals having deposited more circuli on their scales for a given scale length. Fukuwaka and Kaeriyama [28] reported that in an experiment measuring individual growth of juvenile sockeye salmon (*O*. *nerka*), circulus spacing was determined by both the rate of circulus formation and growth of the scale radius. However for the same growth rate, circulus spacing was reported to be wider and circuli deposited less frequently in colder compared with warmer rearing environments [29].

Lack of variation in mean length at age for chum salmon (*O*. *keta*) and sockeye salmon after one year of ocean residence during a 29-year sampling period in the central Bering Sea that was reported by [30] has been interpreted by [12] as providing strong evidence of size-selective mortality during the first year at sea. The implication is that since these fish would have experienced different ocean conditions during their first year of ocean residence, and thus the potential for substantial variation in growth rate, size-selective mortality during the first year of ocean residence would have removed those fish from the population that failed to attain a critical size, and this invariant critical size would then lead to a constant mean size after one year of rearing. However, Fukuwaka et al [31] noted that the data outlined by [24] were obtained by a size-selective gillnet survey, and thus gillnet size selectively may have contributed substantially to an apparent stability in mean length at age. An analysis of variation over a 32-year period for the distance from the scale focus to the first marine annulus was reported for two stocks of Bristol Bay sockeye salmon [32], with scales derived from adults that had returned to spawn. During most of this period, the index displayed a constant mean and variance. However, as noted previously, if size-selective mortality is operating on the juvenile population, then one should observe a shift in the frequency distribution of the scale index towards values associated with larger individuals, but that all adults in the sample should display scale index values observed in the juvenile population. Unfortunately, as noted by Farley et al. [32], attempts were made to collect scales from juvenile sockeye salmon during all surveys, but no comparisons were possible because sample sizes of scales were too small for statistical analyses because of descaling of the juvenile salmon by a mid-water rope trawl. Thus it is unclear if a relatively constant size after one year of ocean rearing is indeed evidence for size-selective mortality, or simply a manifestation of compensatory growth rate. Yamusiishi et al. [33] reported both compensatory growth and size-selective mortality for Bristol Bay sockeye salmon, but the magnitude of size-selective mortality relative to total mortality was uncertain.

Size-selective mortality is often invoked as an important driver of population dynamics of Pacific salmon [6], so much so that the concept has been introduced of the necessity of obtaining a “critical size” by the fall of the first year of ocean rearing in order to ensure survival over the winter [2], a potential second “critical period” [10]. There is no doubt that there can be substantial mortality of Pacific salmon during their first year of ocean residence and as we have documented, size-selective mortality can be observed [14], and in some cases attributed to a single avian predator [34]. However, in order to invoke size selection as an important driver of this mortality, we suggest that it is necessary to demonstrate that size-selective mortality directed towards the smaller members of the population can account for a substantial portion of the observed mortality. With respect to size, a critical size implies that the individuals must attain this size or die; for a size to be defined as “critical,” it must be demonstrated that the proportion of the population failing to attain this size by the specified period can account for the observed mortality. Studies that employ scale characteristics to infer a critical size not only need to show a shift toward larger values of SCSI, but also a concomitant reduction in the variance and range of SCSI and an increase in the skewness and kurtosis of the SCSI values. Additionally, most SCSI values in the adult population should be present in the juvenile population, and that the adult scale classes were a subset of those present in the juvenile population. The actual results of many of the published marine studies where the authors concluded that size-selective mortality coupled with a critical size limit hypothesis was confirmed were actually more supportive of the hypothesis that an unsampled second juvenile population of larger individuals contributed to a portion of the adult sample, producing the results observed in the studies. The critical size hypothesis has been revised such that the word “size” should be interpreted as “condition” [35], and is subject to the same limitations of empirical data support as the critical size hypothesis. Although still likely an oversimplification, we suggest that the two-population scenario is actually more reflective of current understanding of juvenile salmon ocean migration dynamics where relatively “larger” individuals disperse more quickly than relatively “smaller” individuals.

Through a consideration of sampling theory, statistical principles, and simulation, we presented an analytical framework and provided expectations from which contrasts between samples of juveniles and adults may be judged as supporting the critical-size and critical-period hypothesis; an omission of the initial formulation and subsequent studies. Within this framework, as we found no support for the hypothesis after consideration of studies interpreted as supporting the hypothesis, we can only surmise that this failure reflects incorrect conclusions drawn previously about the importance of a critical size and critical period for Pacific salmon survival during the first marine winter.

## Methods

The critical size limit hypothesis requires that salmonid juveniles attain a sufficient size by the end of the first marine summer to be able to survive the subsequent late fall and winter, resulting in those individuals that failed to attain the critical size disappearing from the population. Failure to attain a critical size implies a knife-edge function, whereby individuals failing to attain this critical size die and disappear from the population. If the SCSI in the original juvenile population is normally distributed, and if the critical size hypothesis is valid, then the distribution of SCSI values in the adult population should display a truncated normal distribution such that some portion of the left side (smaller SCSI values) of the juvenile SCSI distribution is absent in the adult SCSI distribution. Theoretically, samples from the adult population should display a larger mean and reduced variance relative to samples from the juvenile population [36], decreased range, and increased skewness and kurtosis [37].

The expected theoretical results were evaluated under the assumption that a population of juvenile salmonids had been sampled and a SCSI value had been measured, with the proviso that the index was normally distributed within a 1,000,000-individual juvenile population with a mean of 30 and a standard deviation of 5. The distribution of SCSI values was simulated via [38]. Initial samples of 50, 200 and 1,000 juveniles were drawn from the population to represent the juvenile samples. The juvenile population was then subjected to size-selective mortality with a critical size limit under the assumption that a critical size had to be obtained in order to survive to the adult stage. We applied size-selective mortality rates with a critical size limit of 20%, 40%, 60%, and 80% to obtain the adult samples, whereby potential adults were discarded from the adult sample if they displayed a SCSI value less than the critical limit (a knife-edge approach to mortality), and the population was sampled until the target sample size was obtained. Ten thousand replicates were simulated independently for each combination of juvenile sample size, adult sample size, and mortality rate. For each replicate, the mean, range, minimum value, maximum value, kurtosis, skewness, and percentage of adults > the largest individual in the juvenile sample were calculated. The 50^th^ percentile of each distribution was used to determine the value of each descriptive statistic. Critical limits for the SCSI were set at 25.79 (20% mortality), 28.74 (40% mortality), 31.27 (60% mortality), and 34.21 (80% mortality). We evaluated trends in the mean, standard deviation, range, minimum SCSI value, maximum SCSI, kurtosis, and skewness via [39], as well as the percentage of individuals in the adult sample that displayed SCSI values greater than the maximum value observed in the corresponding juvenile sample. As SCSI values were considered fixed in the life cycle, then the resultant distributions and trends in descriptive statistics should mimic those observed in actual salmonid populations if indeed size-selective mortality coupled with a knife-edge critical size had occurred, and juvenile and adult samples were derived from the same population.

The final simulation proceeded on the basis that the adult sample could have arisen from sampling two distinct juvenile populations. The first juvenile population was again assumed to be normally distributed with a mean of 30 and a standard deviation of 5. Two states were evaluated for the second juvenile population: mean of 35 with a standard deviation of 5, and mean of 40 with a standard deviation of 5. Adult sample sizes of 50 and 200 individuals were constructed, with juvenile population 1 contributing 90%, 80%, 70%, 60%, 50%, and 40% of the adult sample, with juvenile population 2 contributing 10%, 20%, 30%, 40%, 50%, and 60%, respectively. The 50^th^ percentile of each of 10,000 distributions was used to determine the value of each descriptive statistic. Trends in the mean, standard deviation, range, minimum SCSI value, maximum SCSI, kurtosis, and skewness of the adult sample were evaluated via [38], as well as the percentage of individuals in the adult sample that displayed SCSI values greater than the maximum value observed in the corresponding juvenile sample from population 1. No size-selective mortality was applied in this simulation, only differing contributions from two juvenile populations where the mean SCSI values differed between populations. Data for simulations outlined in the study are available via DRYAD doi identified as: data package title: Data from: Validity of inferring size-selective mortality and a critical size limit in Pacific salmon from scale circulus spacing. https://doi.org/10.5061/dryad.n560cd7. Files: SimulatedStatistics-KnifeEdgeMortality.csv and SimulatedStatistics-TwoSamples.csv.
